# Clinical spectrum and the comorbidities of Dravet syndrome in Taiwan and the possible molecular mechanisms

**DOI:** 10.1038/s41598-021-98517-4

**Published:** 2021-10-12

**Authors:** Chia-Hsuan Huang, Pi-Lien Hung, Pi-Chuan Fan, Kuang-Lin Lin, Ting-Rong Hsu, I-Jun Chou, Che-Sheng Ho, I-Ching Chou, Wei-Sheng Lin, Inn-Chi Lee, Hueng-Chuen Fan, Shyi-Jou Chen, Jao-Shwann Liang, Yi-Fang Tu, Tung-Ming Chang, Su-Ching Hu, Lee-Chin Wong, Kun-Long Hung, Wang-Tso Lee

**Affiliations:** 1 Division of Pediatric Neurology, Department of Pediatrics, National Taiwan University Hospital Yunlin Branch, Yunlin County, Taiwan; 2 Department of Pediatric Neurology, Chang Gung Memorial Hospital-Kaohsiung, Kaohsiung, Taiwan; 3 Department of Pediatrics, National Taiwan University Hospital and National Taiwan University College of Medicine, Taipei, Taiwan; 4 Division of Pediatric Neurology, Chang Gung Children’s Hospital and Chang Gung Memorial Hospital, Chang Gung University College of Medicine, Taoyuan, Taiwan; 5 Department of Pediatrics, Taipei Veterans General Hospital, Taipei, Taiwan; 6 Department of Pediatrics, Mackay Memorial Hospital, Taipei, Taiwan; 7 Division of Pediatrics Neurology, China Medical University Children’s Hospital, Taichung, Taiwan; 8 Institute of Medicine, School of Medicine, Chung Shan Medical University, Taichung, Taiwan; 9 Department of Pediatrics, Tungs’ Taichung Metroharbor Hospital, Taichung, Taiwan; 10 Department of Pediatrics, Tri-Service General Hospital, National Defense Medical Center, Taipei, Taiwan; 11 Department of Pediatrics, Far Eastern Memorial Hospital, New Taipei City, Taiwan; 12 Department of Pediatrics, National Cheng Kung University Hospital, College of Medicine, National Cheng Kung University, Tainan, Taiwan; 13 Department of Pediatric Neurology, Changhua Christian Children’s Hospital, Changhua, Taiwan; 14 Department of Pediatrics, Cathay General Hospital, Taipei, Taiwan; 15 Department of Pediatrics, Fu-Jen Catholic University Hospital, Fu-Jen Catholic University, New Taipei City, Taiwan; 16 Department of Pediatric Neurology, National Taiwan University Children’s Hospital, 8, Chung-Shan South Road, Taipei, 100 Taiwan

**Keywords:** Neurology, Epilepsy

## Abstract

Dravet syndrome (DS) is an uncommon epilepsy syndrome that may negatively affect the patients and their caregivers. However, reliable and valid measures of its impact on caregivers and the characteristics of patients with DS in Taiwan are lacking. This study aimed to describe the characteristics of patients with DS and concerns of their caregivers and establish a baseline frequency of disease characteristics using a cross-sectional survey in Taiwan. We assessed the caregivers of patients with DS using an online anonymous questionnaire. The seizure frequency decreased with age, although lacking statistical significance. Vaccines show no influence on the condition of patients with DS. Our findings revealed the highest impact on the domains affecting the caregivers’ daily life, including additional household tasks, symptom observation, further medical plan, and financial issues. Caregivers also expressed concerns regarding the lack of independence/constant care, seizure control, speech/communication, and impacts on siblings because of long-term care of the patients in parents’ absence. Our findings highlight the significant effects of caring for a child with DS on the lives of their caregivers in Taiwan; these findings will help raise awareness regarding the needs of these families. Furthermore, we discussed the possible pathophysiological mechanisms of associated comorbidities.

## Introduction

Dravet syndrome (DS), also known as severe myoclonic epilepsy of infancy, is a rare and devastating epilepsy syndrome. The prevalence rate is estimated to be approximately 1 in 20,000 to 1 in 40,000 children^1–3^. The associated mutations of *SCN1A* have been reported in 75% of patients with DS. Patient characteristics in DS include frequently prolonged hemi-convulsion, developmental delay, speech impairment, and other comorbidities such as ataxia, circadian rhythm disorder, impaired sleep quality, and autistic-like social interaction deficits^4^. DS is frequently accompanied with a wide range of triggering factors, such as fever, infections, hot-water bath, and photosensitivity. Although DS is usually pharmacoresistant, a trend toward less severe epilepsy with worsening cognitive impairment is usually observed after the age of 5 years^5^.

Although previous studies have shown no significant difference in the clinical and cognitive outcomes, most parents were concerned regarding vaccination-related seizures^6–8^. Owing to the limited knowledge about the frequency of seizures following vaccination, the misconception regarding vaccination-related side effects and reduced vaccination coverage are still noted among numerous families caring for patients with DS^8^.

We aimed to describe the characteristic features of patients with DS and the concerns of their caregivers and establish a baseline frequency of disease characteristics using a cross-sectional survey in Taiwan. The data may help researchers and clinicians to conduct additional studies and further understand this refractory epilepsy and the significant issues encountered by the patients and their families. In addition, we discussed the possible pathophysiological molecular mechanisms related to DS-associated comorbidities.

## Results

### Demographics

**Figure 1:**
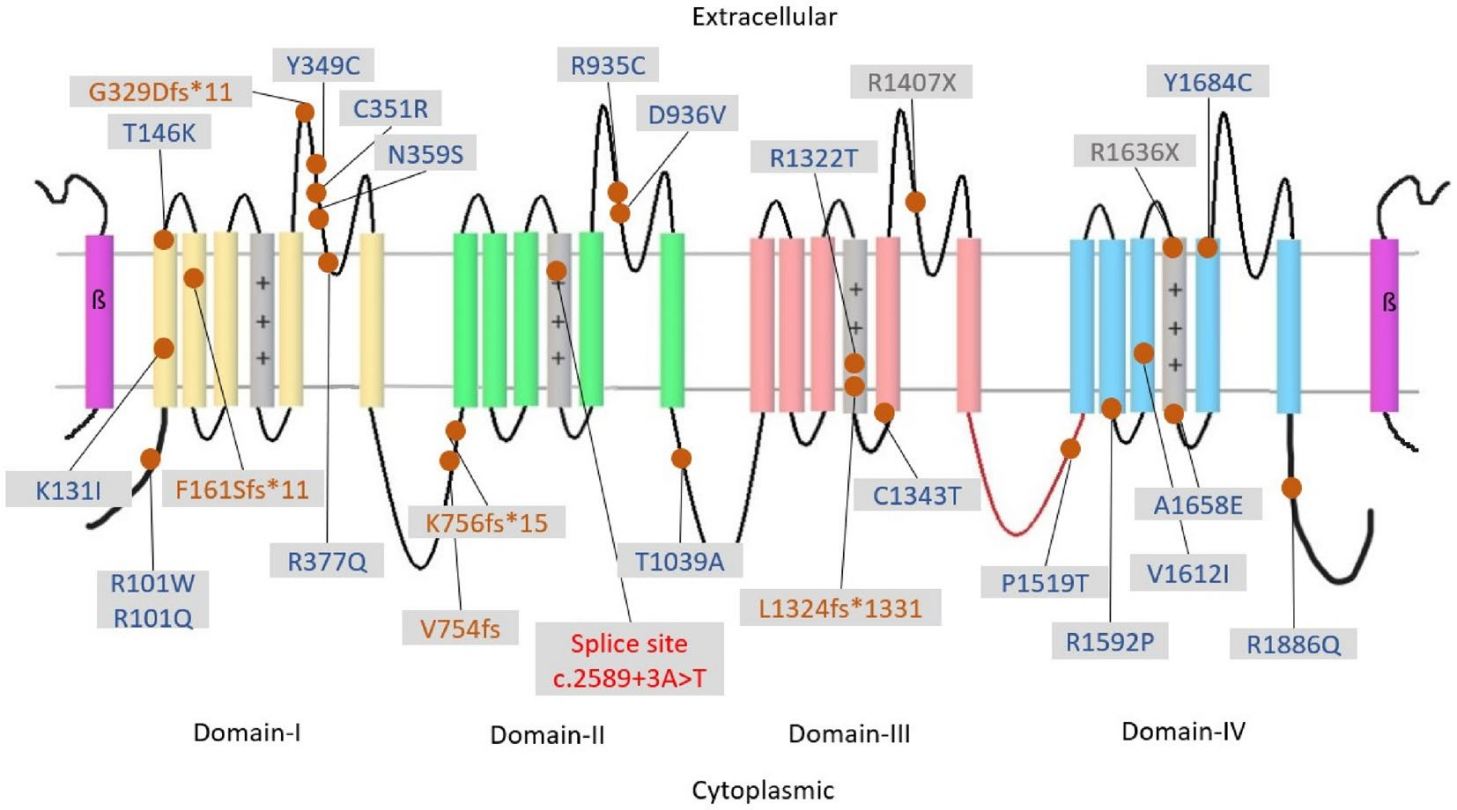
Schematic representation of sodium channel type-1 (Na_v_1.1) mutations in our study. The *SCN1A* alpha unit has four domains (I–IV). Each domain includes six transmembrane segments (S1–S6). Inactivation gate (red line); voltage sensor (gray column). Other two deletion mutations are not marked: (1) 2q24.3q31.1 deletion; (2) microdeletion 2q chromosome. Missense (blue); frameshift (brown); nonsense (gray); splice site (red).

We identified 38 patients with DS, all of whom had a confirmed mutation in *SCN1A*. In total, 32 patients were aware of the correct mutation data: 19 missense mutations in 21 patients, 2 nonsense mutations in 2 patients, 1 splice-site mutation in 1 patient, 5 frameshift mutations in 6 patients, and 2 chromosome deletions in 2 patients (Fig. 1). Patient age was 1–28 (mean ± standard deviation [SD]: 10.5 ± 6.3) years. In total, 16 patients (42.1%) were female (Table 1). Regarding the language and ambulation evaluation, excluding patients aged < 2 years, 51% could speak a clear and correct sentence and 78% could ambulate without assistance.

**Table 1:** Demographics data of 38 patients with DS. Data are presented as mean ± SD.

Patient data	N (%)	Patient data	N (%)
**Age**		**Seizure pattern**	
Infants (0–1 year)	1 (2.6)	Any seizures	38/38 (100)
Preschoolers (2–5 years)	7 (18.4)	Generalized tonic–clonic	25/38 (66)
Middle-childhood patients (6–11 years)	18 (47.3)	Atypical absence	14/38 (37)
Adolescents (12–17 years)	7 (18.4)	Focal	11/38 (29)
Adults (≥ 18 years)	5 (13.1)	Myoclonus	6/37 (16)
Participant age (years)	10.5 ± 6.3	Epileptic spasms	1/38 (3)
Female	16 (42)		
Male	21 (58%)		
*SCN1A* mutation	38 (100%)		
First seizure age (months)	9.5 ± 16.1		

### Seizures

**Figure 2:**
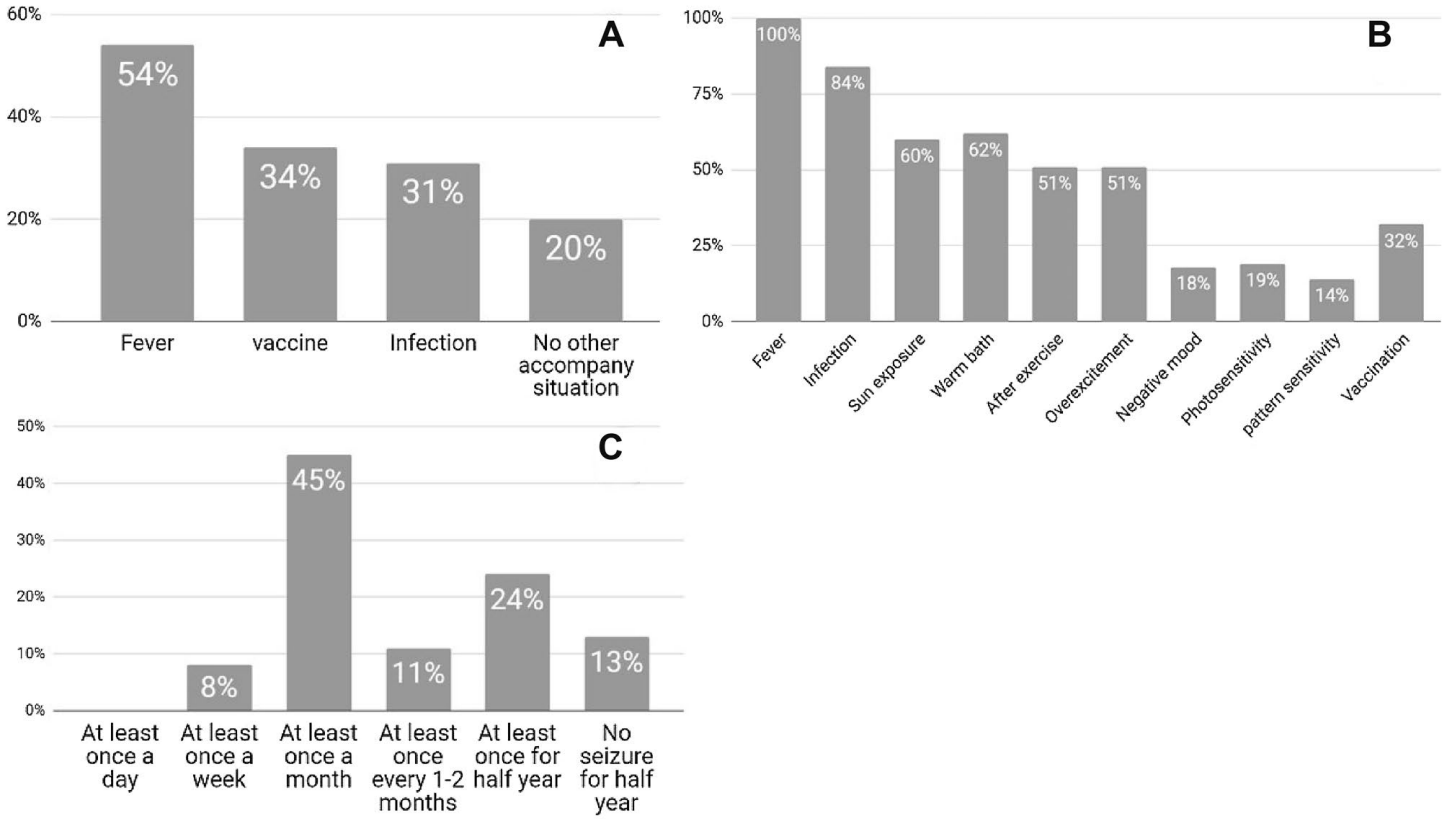
Questionnaire findings regarding seizure evaluation. (**A**) Situations accompanying the first seizure occurrence. (**B**) Situations accompanying the subsequent seizures. (**C**) Seizure frequency.

The mean age of the patients enrolled at the first seizure was 9.5 ± 16.1 months. All patients had seizures during the clinical course. The generalized tonic–clonic, absence, and focal seizures were the most common at first observation and occurred in 66%, 37%, and 29% of the patients, respectively (Table 1). These seizures were frequently induced by fever (54%) (Fig. 2A), and caregivers reported that fever, infection, sun exposure, hot-water bath, exercise, and overexcitement were the most common factors triggering subsequent seizures (Fig. 2B). The incidence of these triggering factors may be > 50%. The occurrence of first seizure due to vaccination was noted in 34% of patients with DS. Vaccination-triggered seizures were observed in 32% of the patients.

Photo- and pattern sensitivities also were triggering factors for seizure. These sensitivities may be observed across different age groups and may not disappear with aging.

Seizures mostly occurred weekly and monthly in 53% patients (Fig. 2C). No considerable differences were observed among different age groups: infants, 1/1 (100%); preschoolers (2–5 years), 4/7 (57%); middle-childhood patients (6–11 years), 10/18 (56%); adolescents (12–17 years), 4/7 (57%); and adults (≥ 18 years), 1/5 (20%). Although no significant difference was noted (P = 0.329), the seizure frequency decreased after the age of 18 years. The possible reasons for the high incidence of seizures for patients aged > 12 years may be related to the delayed diagnosis of DS in this age group. In total, 3/7 patients in this age group were diagnosed with DS after the age of 12 years. All of them experienced frequent seizures.

The yearly frequency of emergency department visit was 2.0 ± 1.28 for patients aged 0–5 years, 1.88 ± 2.37 for those aged 6–11 years, and 0.50 ± 0.76 for those aged 12–18 years. Except for one patient with 30 visits per year, the yearly frequency of emergency department visit for those aged > 18 years was 0.25 ± 0.50. A significant decrease in visit frequency was observed in patients aged 12–17 and those aged ≥ 18 years (P < 0.01). The admission frequency was 1.75 ± 1.49 for patients aged 0–5 years, 1.18 ± 1.70 for those aged 6–11 years, 0.38 ± 0.52 for those aged 12–18 years, and 0.25 ± 0.50 for those aged > 18 years. A significant decrease in admission frequency was also observed in patients aged > 12 years (P < 0.05).

### Vaccination

When we compared the vaccination-proximate (seizure attack within < 48 h after vaccination) and vaccination-distant (seizure attack within ≥ 48 h after vaccination) groups, no significant difference was observed between the two groups. No significant difference was present in language, ambulation, seizure characteristics (i.e., first seizure onset, seizure-triggering factors except vaccine, seizure pattern, and seizure frequency) and the number of antiepileptic drugs (AEDs) used.

### Comorbidities

All questionnaires were filled out by 100% of the caregivers (Table 2). With the exception of nocturnal seizures, a slight variation was recorded in sleep issues among the different age groups.

Bradycardia and tachycardia were reported in 9% and 3% of the patients, respectively. One patient had a history arrhythmia, and 3 out of 36 patients reported abnormalities or changes in the heart structure. Of these patients, one had ventricular septal defect, one had trivial tricuspid regurgitation, and one withheld their cardiac anomaly details.

Behavioral and psychiatric issues were commonly reported, and most (61%) had a complaint about attention-deficit disorder or attention-deficit hyperactivity disorder. Other psychiatric symptoms such as difficulty with impulse control and autistic-like traits were also noticed in 39% and 31% of the patients, respectively. Of the patients, 42% visited pediatric psychiatric clinics for evaluation. Anxiety and psychosis were recorded in one-third of the patients with DS.

Regarding musculoskeletal issues, hypotonia (50%) was relatively common in the patients’ early childhood. Broken bones (24%) and scoliosis (19%) increased with age and were more prevalent in middle-childhood to adult patients than in infant to preschooler patients.

Constipation (47%) was another common issue. About one-third of patients with DS had an appetite disturbance and a frequent/chronic urinary tract infection.

Drowsiness, cognition problem, and unsteady gait were the most common drug-related side effects and occurred in 46%, 47%, and 49% of the patients, respectively. An unsteady gait also occurred due to DS.

**Table 2:** Frequencies of the most commonly reported comorbidities.

Issue reported	n/N (%)	Issue reported	n/N (%)
**Psychiatric issues**		**Sleep issues**	
Autistic-like traits	11/36 (31)	Sleep disorders	11/38 (29)
Difficulty with impulse control	15/38 (39)	Nocturnal seizures	18/35 (51)
ADD or ADHD	23/38 (61)	Irregular sleep schedule	6/38 (16)
Anxiety	8/37 (22)	Premature awakening	7/37 (19)
Psychosis	6/31 (19)	Sleep apnea	3/37 (8)
Depression	1/35 (2)	**Cardiac**	
Pediatric psychiatry clinic	16/38 (42)	Tachycardia	3/35 (9)
**Urinary tract and bowel**		Bradycardia	1/35 (3)
Slow digestion	5/38 (13)	Arrhythmia	1/36 (3)
Appetite disturbance	11/38 (29)	Abnormalities of heart	3/36 (8)
Constipation	18/38 (47)	**Orthopedic/movement**	
GERD	6/37 (16)	Hypotonia	17/34 (50)
Diarrhea	4/38 (11)	Hypertonia	3/34 (9)
Frequent urinary tract infections	11/38 (29)	Broken bones	9/38 (24)
Nephrocalcinosis	1/38 (3)	Scoliosis	7/37 (19)
**Hematologic issues**		Hip dysplasia	3/35 (9)
Thromobocytopenia	3/36 (8)	**Drug side effect**	
Vitamin D deficiency	2/34 (6)	Drowsiness	17/37 (46)
Iron deficiency/anemia	1/35 (3)	Cognition problem	16/34 (47)
Significant hair loss	6/36 (17)	Unsteady gait	17/35 (49)
Neutropenia	2/36 (6)		

*ADD* attention-deficit disorder, *ADHD* attention-deficit hyperactivity disorder, *GERD* gastroesophageal reflux disease.

### Medication survey

The sixth most common daily medications used by the patients were clobazam (68%), valproic acid (66%), levetiracetam (55%), topiramate (29%), stiripentol (26%), and clonazepam (18%). The use of contraindicated medications, including lamotrigine (11%), carbamazepine (3%), and oxcarbazepine (24%), was also reported. The survey did not distinguish between medications used in prediagnosis and postdiagnosis.

In most patients, multiple AEDs were needed and 78% needed > 3 drugs for seizure control. All (12/12) of the responders with children aged ≥ 12 years reported having to use > 3 AEDs. Of the patients, 5% (2/38) and 16% (6/38) used one and two drugs for seizure control, respectively.

### Caregiver issues and family dynamics

Nearly half of the caregivers (47%) reported having suffered from depressed mood, but we did not record whether they had received further help. When evaluating the caregiver burden scale in each domain, approximately three-quarters of the caregivers reported a moderate or greater difficulty in performing additional household tasks (79%), observing and reporting symptoms (77%), and seeking further medical plans (76%). The rest of the items in the questionnaire were regarding financial issues (66%), medical or nursing treatments (66%), medication use (63%), patient care (58%), and mobility problems (50%). When asked to rank their top three concerns in an open response, caregivers highlighted the lack of independence (61%), seizure control (58%), speech and communication challenges (50%), and impacts on siblings because of long-term care of patients with DS in the absence of parents (50%) (Supplementary Tables 1, 2).

## Discussion

In this cross-sectional cohort, we collected data on patients with DS from their caregivers in Taiwan, improving our understanding of the impact the conditions these patients have on their caregivers. Specific seizure-triggering factors of DS must be avoided. In our study, hyperthermia was the most significant triggering factor, in which is consistent with the findings of other studies. Therefore, patients should avoid the environment or conditions of hyperthermia, such as overexcitement, overexertion, sun exposure, and hot-water bath^9^. Family members of the patients should also be informed to seek medical assistance whenever the patients experience hyperthermia. We also noticed that photo- and pattern sensitivities triggered seizures, similar to the result of the study by Villas et al. (2017)^10^.

In our study, we discovered vaccine-related seizures in 12 (34%) of 35 patients in our cohort. This finding is consistent with that of previous studies, which showed that one-third of patients with DS developed seizures after vaccination^7,8,11^. No significant difference was observed in language, ambulation, or seizure characteristics between patients with and without vaccine-related seizures. Therefore, based on the results of previous studies and the present study, no difference was noted in terms of the clinical outcomes, subsequent seizure frequency, and genetic etiology when comparing vaccination-proximal and vaccination-distant groups (Table 3). Thus, vaccination should not be withheld from patients with DS and all clinicians should provide families with accurate and sufficient information before vaccinating the patients.

**Table 3:** Literature review regarding vaccination-related seizures in patients with DS.

Study	Present study	Wong et al. Pediatr Neurol 2016^8^	Tro-Baumann et al. Epilepsia 2011^11^	McIntosh et al. Lancet Neurol 2010^7^
Country	Taiwan	Hong Kong	Germany and Austria	Austria
Numbers	38	54	70	40
Ethnic origin	100% Chinese	98% Chinese	Unspecified	Unspecified
Percentage of vaccination-related seizures	34%	31.5%	27%	30%
Significance of *SCN1A* mutations	100%	83.2%(45/54)	100%	100%
Major findings	No statistically significant difference in language, ambulation, or seizure characteristics	No difference between the clinical outcome and subsequent seizure development. Absence seizure and status epilepticus are more likely to occur in vaccination-proximate group	58% of patients with Dravet syndrome had vaccination-related seizure as first clinical manifestation	Vaccination should not be held due to no differences in intellectual outcome, subsequent seizure type, or SCN1A mutation type when comparing vaccination-distant with vaccination-proximal group

Previous studies have reported that seizure frequency decreases with age, which is independent of the type of *SCN1A* mutation^12–15^. We also observed a tendency of decrease in seizure frequency with age, although this result showed no statistical significance. This could be because of the low number of patients aged > 12 years and delayed DS diagnosis in many patients aged 12–17 years who had more frequent seizures. The emergency department visits and admission frequency decreased after the age of 12 years, and these findings were consistent with those of previous studies. In a previous study, fever sensitivity persisted in adolescent and adult patients with DS but exhibited less influence^14^.

Previous studies on Dravet mouse models have demonstrated that seizure susceptibility in DS is caused by the reduced sodium currents and electrical excitability of gamma-aminobutyric acid-ergic (GABAergic) interneurons, which may lower the seizure threshold^16,17^. The first-line AED therapy for patients with DS include valproic acid and clobazam, and the second-line therapy may include topiramate, stiripentol, and a ketogenic diet^18^. As shown in Table 4, valproic acid was the most commonly used AED. Clobazem, topiramate, and stiripentol were also used frequently. By contrast, levetiracetam was the third most commonly used AED in the treatment of patients with DS in Taiwan.

**Table 4:** Review of real-world evidence on the medicine utilization of patients with DS in the literature.

Study	Present study	Schubert-Bast et al. Epilepsy Behav 2019^19^	Villas et al. Epilepsy Behav 2017^10^	Lagae et al. Dev Med Child Neurol 2018^20^	Aras et al. Epilepsy Behav 2015^21^
Year of survey	2019/2020	2017–2018	2016	2016	2014
Country	Taiwan	Germany	Worldwide	Worldwide	Europe
Numbers	38	93	159	584	274
Age (years)	Mean: 10.5	Mean: 10.1	Median: 7–10	Mean: 10.6	Median: 4–8
Most used AEDs	1. Clobazam (68%)	1. Valproate (66%)	1. Valproate (89%)	1. Valproate (76%)	1. Valproate (86%)
	2. Valproic acid (66%)	2. Bromide (44%)	2. Levetiracetam (87%)	2. Clobazam (53%)	2. Clobazam (55%)
	3. Levetiracetam (55%)	3. Clobazam (41%)	3. Clobazam (82%)	3. Stiripentol (47%)	3. Topiramate (44%)
	4. Topiramate (29%)	4. Stiripentol (35%)	4. Topiramate (79%)	4. Topiramate (34%)	4. Stiripentol (42%)
	5. Stiripentol (26%)	5. Topiramate (15%)		5. Bromide (10%)	5. Levetiracetam (22%)
	6. Clonazepam (18%)				

*AED* antiepileptic drug.

Drowsiness, cognition problem, and unsteady gait were the most common side effects of AEDs observed in our study. By contrast, hematologic side effects such as thrombocytopenia, neutropenia, or anemia exhibited no significance. Nephrocalcinosis due to topiramate was noted 3% of the patients, which was similar to that observed in another study^10^. Appetite disturbance and constipation were also noted in our patients, and this could be due to AEDs or DS itself.

In our study, the characteristic symptoms of DS included nocturnal seizures, hypotonia, drowsiness, cognition problem, unsteady gait, constipation, and psychiatric issues such as ADD or ADHD, which are similar to the findings of previous studies^10,22^. In our study, caregivers reported nocturnal seizure among 51% of the patients, the same as that reported in a previous study^23^; this value was lower than that of another study, which reported nocturnal seizure in 77% of patients^10^. These results indicate that nocturnal seizures are a major concern for most caregivers. Recently, awareness regarding the association between DS with *SCN1A* mutations and heart-rate abnormalities has increased. Heart-rate abnormalities leading to sudden death may be a major concern for most caregivers^24^. Although cardiac arrhythmia was noted in one of our patients, none of them suddenly died due to cardiac problems.

We briefly discuss the possible pathophysiological molecular mechanisms leading to different DS-associated comorbidities in the past. DS is caused mainly by a heterozygous loss-of-function mutation in *SCN1A*, which encodes voltage-gated Na_v_1.1 channel. The Nav1.1 channel is a member of the family of voltage-sensitive sodium channels, including Na_v_1.1, Na_v_1.2, Na_v_1.3, Na_v_1.6, and Na_v_1.7^25,26^. Because Na_v_1.1 channel expression is extremely low in neonates, other subunits such as Na_v_1.2 and Na_v_1.3 may compensate for the reduced Na_v_1.1 expression in the early stage of brain development^26^. Na_v_1.1 level increases overtime in brain maturation^25^. However, in Dravet syndrome mouse model, failure of increased expression in function of Na_v_1.1 channels during physiologically decreased expression in Na_v_1.3 channels may lead to intractable seizures and various comorbidities, such as ataxia, sleep disorders, and autistic-like behaviors, and spatial learning and memory defects^25,26^.

Electrophysiological studies in the past showed that Na_v_1.1 channels may play an important role in the excitability of Purkinje neurons of the cerebellum, resulting in the activation of sodium currents and sustained action potential firing^27^. In mutant mouse models, the loss of these channels may cause the dysfunction of cerebellar Purkinje neurons, leading to ataxia^27^.

Patients with DS frequently have sleep disorders, including impaired sleep duration and increased nocturnal seizures^28^. In DS mouse model, mutation of Na_v_1.1 channel in forebrain cause impaired action potential firing in reticular nucleus of the thalamus GABAergic interneurons, leading to sleep disorders^29^. In DS, patients may also have a circadian rhythm disturbance, affecting their sleep–wake cycle^28^. Although our result did not reveal significant findings related to this topic, in the DS mouse model, it was shown to have an abnormal circadian cycle length and impaired light-induced shifts in sleep–wake cycle^28^. In another study with heterozygous *Scn1a*^+/−^ mice, the reduction of Na_v_1.1 activity was suggested to impair the suprachiasmatic nucleus of the hypothalamus, which is the primary site of the circadian clock^30^. These studies suggested that the decreased GABAergic transmission plays a role in circadian defect^30^. Therefore, sleep disorders in DS may be treated with the improvement of GABAergic neurotransmission^30^.

Patients with DS also show autistic-like behaviors^10^. DS mice also had significant social-interaction deficits^31^. The deficit may arise from specific disturbances in the Na_v_1.1 channel in the forebrain inhibitory neurons rather than the epileptic activity itself^31^. Therefore, the treatment with low-dose clonazepam may improve the autistic-like behaviors in DS mice^31^. Furthermore, in 2015, Rubinstein et al. also showed that GABAergic interneurons may include parvalbumin-(PV+) or somatostatin-expressing (SST+) interneurons^32^. The disturbance in the Na_v_1.1 channel in PV+ interneurons may cause social-interaction deficits. However, the disturbance in the Na_v_1.1 channel in SST+ interneuron may cause hyperactivity. By contrast, the synergistic effects of PV+ and SST+ interneurons impaired the long-term spatial memory^32^. These studies demonstrated that autistic-like phenotypes and spatial learning deficits may result from the decreased Na_v_1.1 activity in GABAergic interneurons in the hippocampus and cortical interneurons^17,31,33^.

Sudden unexpected death in epilepsy (SUDEP) is one of the common causes of death in patients with drug-resistant epilepsies and is also the possible cause of death in DS; however, the pathophysiological mechanisms leading to SUDEP remain unknown^23,34^. Although we did not document these events in our study, recent studies indicated that SUDEP is caused by parasympathetic hyperactivity following hyperthermia-induced tonic–clonic seizures. It has been demonstrated to cause severe bradycardia and death in an Scn1a^+/−^ mouse model^34^. The alterations in neuronal excitability and cardiac electrophysiology in ventricular myocytes result in the arrhythmogenesis and SUDEP^35^. The reductions in Na_v_1.1 expression may also indirectly affect the Na_v_1.5 channel and cardiac functions^35^, leading to cardiac issues.

**Figure 3:**
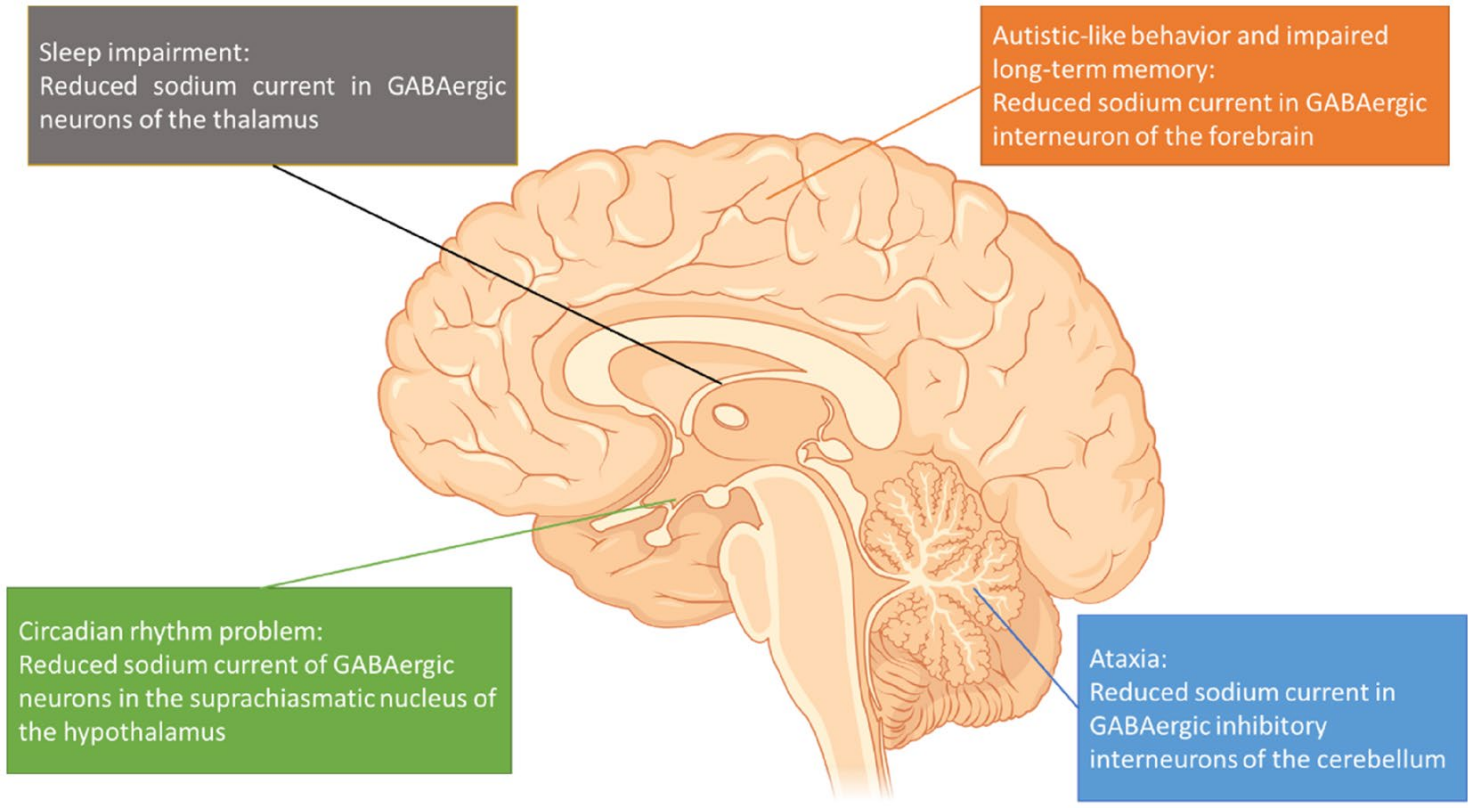
Schematic representation of the possible mechanisms of DS-associated comorbidities (partially created with https://biorender.com).

Therefore, regaining the impaired GABAergic neurotransmission may improve both the seizure control and function of the prefrontal cortex to cerebellar networks^31,32,36^ (Fig. 3).

Several studies have focused on the caregivers of patients with DS owing to the different aspects of stress. Therefore, a multidisciplinary team may be needed to care for the patients. In our study, caregivers viewed additional household tasks, symptom observation, further medical plan, and financial issues as significant factors. Although a public health insurance system exists in Taiwan, our findings indicate that caregivers in Taiwan are still concerned regarding the medical expenses and environment other than their patient’s medical condition. This finding may be influenced by the medical system and medical security provided in each country or region. A cohort study conducted at Children’s Hospital Colorado showed that caregivers suffered from emotional exhaustion and anxiety related to “fear of the next seizure” and “the seizure that kills my child.” Furthermore, they need to quit their jobs or careers to take care of their children because of the severity of the neurological symptoms and comorbidities^37^. In another study, persistent severe seizures, accompanied with developmental, cognitive, behavioral, and sleep disorders, have also been reported to increase the caregivers’ burden^28^. Most caregivers are also concerned about sleep deprivation, emotional problems, social-interaction deficits, and economic burdens^38^.

The caregivers in this study ranked their top three major concerns in the future, which include the lack of independence/constant care, seizure control, speech and communication problems, and impacts on siblings (long-term care in the absence of the patient’s parents). In a previous study, caregivers ranked their top four concerns, which included speech and communication challenges, impact on patients’ siblings, cognitive and developmental delay, and behavioral disorders such as violence and autistic traits^10^. Therefore, awareness about caregivers’ needs and additional psychological support has become increasingly important to relieve the burden of the caregivers, thereby improving their physical and emotional well-being.

Our study has several limitations. Not all patients with DS from Taiwan were enrolled in our study. In addition, the most common seizure pattern in adolescents and adults was generalized tonic–clonic seizures and they were mostly nocturnal and existed in clusters^14,39^. However, we did not record the serial seizure changes. Furthermore, owing to the lack of blood-report data of our patients, we could not establish any positive association of broken bones and scoliosis with DS or other etiologies such as vitamin D deficiency.

In conclusion, comorbidities are very common in patients with DS, and they are associated with the involvement of different brain regions. Therefore, a detailed evaluation of patients with DS for the possible association of different comorbidities may direct neurologists to provide accurate treatments in addition to that required for seizures.

## Methods

### Survey design

We recruited the caregivers of patients with a diagnosis of DS. All cases had been diagnosed and actively followed up by a pediatric neurologist in Taiwan. An online questionnaire regarding demographic data, gene mutation, clinical features, vaccine use, and the impact on the family was designed, and the caregivers and their doctors were requested to fill out the form. Participation in this online questionnaire study was voluntary, and data were collected anonymously. Permission to use deidentified data was obtained before participation, and each survey included a demographic session and content-related sections about the characteristics, possible comorbidities, medications and efficacy, and caregiver/family dynamics. The responses included lists, closed multiple-choice questions, and open responses. This study was approved by the ethical committee of National Taiwan University Hospital. Informed consent was obtained online from the responders or parents/legally authorized representatives of patients aged < 18 years. All procedures were performed in accordance with relevant guidelines and regulations.

We also assessed the period between the first seizure and the previous vaccination. We defined two groups based on seizure occurrence time, the vaccination-proximate (seizure within < 48 h after vaccination) and vaccination-distant (seizure within ≥ 48 h after vaccination) groups, as described in previous studies^7,8^.

### Statistical analysis

Data are presented as mean ± SD. Statistical comparisons between the groups were performed using Chi-square tests, and p-values of < 0.05 were considered significant. Statistical analysis was performed using IBM SPSS Statistics version 22 (IBM Corp., Armonk, NY, USA).

## Supplementary Information

Supplementary Tables.

## References

[1] Brunklaus A, Ellis R, Reavey E, Forbes GH, Zuberi SM (2012). Prognostic, clinical and demographic features in SCN1A mutation-positive Dravet syndrome. Brain.

[2] Hurst DL (1990). Epidemiology of severe myoclonic epilepsy of infancy. Epilepsia.

[3] Yakoub M, Dulac O, Jambaque I, Chiron C, Plouin P (1992). Early diagnosis of severe myoclonic epilepsy in infancy. Brain Dev..

[4] Catterall WA (2018). Dravet syndrome: A sodium channel interneuronopathy. Curr. Opin. Physiol..

[5] Dravet C (2011). The core Dravet syndrome phenotype. Epilepsia.

[6] Zamponi N, Passamonti C, Petrelli C, Veggiotti P, Baldassari C, Verrotti A, Capovilla G, Viri M, Coppola G, Vignoli A (2014). Vaccination and occurrence of seizures in SCN1A mutation-positive patients: A multicenter Italian study. Pediatr. Neurol..

[7] McIntosh AM, McMahon J, Dibbens LM, Iona X, Mulley JC, Scheffer IE, Berkovic SF (2010). Effects of vaccination on onset and outcome of Dravet syndrome: A retrospective study. Lancet Neurol..

[8] Wong PT, Wong VC (2016). Prevalence and characteristics of vaccination triggered seizures in Dravet syndrome in Hong Kong: A retrospective study. Pediatr. Neurol..

[9] Wirrell EC, Nabbout R (2019). Recent advances in the drug treatment of Dravet syndrome. CNS Drugs.

[10] Villas N, Meskis MA, Goodliffe S (2017). Dravet syndrome: Characteristics, comorbidities, and caregiver concerns. Epilepsy Behav..

[11] Tro-Baumann B, von Spiczak S, Lotte J, Bast T, Haberlandt E, Sassen R, Freund A, Leiz S, Stephani U, Boor R (2011). A retrospective study of the relation between vaccination and occurrence of seizures in Dravet syndrome. Epilepsia.

[12] Jansen FE, Sadleir LG, Harkin LA, Vadlamudi L, McMahon JM, Mulley JC, Scheffer IE, Berkovic SF (2006). Severe myoclonic epilepsy of infancy (Dravet syndrome): Recognition and diagnosis in adults. Neurology.

[13] Rilstone JJ, Coelho FM, Minassian BA, Andrade DM (2012). Dravet syndrome: Seizure control and gait in adults with different SCN1A mutations. Epilepsia.

[14] Darra F, Battaglia D, Dravet C, Patrini M, Offredi F, Chieffo D, Piazza E, Fontana E, Olivieri G, Turrini I (2019). Dravet syndrome: Early electroclinical findings and long-term outcome in adolescents and adults. Epilepsia.

[15] Akiyama M, Kobayashi K, Yoshinaga H, Ohtsuka Y (2010). A long-term follow-up study of Dravet syndrome up to adulthood. Epilepsia.

[16] Yu FH, Mantegazza M, Westenbroek RE, Robbins CA, Kalume F, Burton KA, Spain WJ, McKnight GS, Scheuer T, Catterall WA (2006). Reduced sodium current in GABAergic interneurons in a mouse model of severe myoclonic epilepsy in infancy. Nat. Neurosci..

[17] Ogiwara I, Miyamoto H, Morita N, Atapour N, Mazaki E, Inoue I, Takeuchi T, Itohara S, Yanagawa Y, Obata K (2007). Nav1.1 localizes to axons of parvalbumin-positive inhibitory interneurons: A circuit basis for epileptic seizures in mice carrying an Scn1a gene mutation. J. Neurosci..

[18] Wirrell EC (2016). Treatment of Dravet syndrome. Can. J. Neurol. Sci..

[19] Schubert-Bast S, Wolff M, Wiemer-Kruel A, von Spiczak S, Trollmann R, Reif PS, Pritchard C, Polster T, Neubauer BA, Mayer T (2019). Seizure management and prescription patterns of anticonvulsants in Dravet syndrome: A multicenter cohort study from Germany and review of literature. Epilepsy Behav..

[20] Lagae L, Brambilla I, Mingorance A, Gibson E, Battersby A (2018). Quality of life and comorbidities associated with Dravet syndrome severity: A multinational cohort survey. Dev. Med. Child Neurol..

[21] Aras LM, Isla J, Mingorance-Le Meur A (2015). The European patient with Dravet syndrome: Results from a parent-reported survey on antiepileptic drug use in the European population with Dravet syndrome. Epilepsy Behav..

[22] Gataullina S, Dulac O (2017). From genotype to phenotype in Dravet disease. Seizure.

[23] Skluzacek JV, Watts KP, Parsy O, Wical B, Camfield P (2011). Dravet syndrome and parent associations: The IDEA League experience with comorbid conditions, mortality, management, adaptation, and grief. Epilepsia.

[24] Delogu AB, Spinelli A, Battaglia D, Dravet C, De Nisco A, Saracino A, Romagnoli C, Lanza GA, Crea F (2011). Electrical and autonomic cardiac function in patients with Dravet syndrome. Epilepsia.

[25] Cheah CS, Westenbroek RE, Roden WH, Kalume F, Oakley JC, Jansen LA, Catterall WA (2013). Correlations in timing of sodium channel expression, epilepsy, and sudden death in Dravet syndrome. Channels (Austin).

[26] Brunklaus A, Zuberi SM (2014). Dravet syndrome—From epileptic encephalopathy to channelopathy. Epilepsia.

[27] Kalume F, Yu FH, Westenbroek RE, Scheuer T, Catterall WA (2007). Reduced sodium current in Purkinje neurons from Nav1.1 mutant mice: Implications for ataxia in severe myoclonic epilepsy in infancy. J. Neurosci..

[28] Nolan KJ, Camfield CS, Camfield PR (2006). Coping with Dravet syndrome: Parental experiences with a catastrophic epilepsy. Dev. Med. Child Neurol..

[29] Kalume F, Oakley JC, Westenbroek RE, Gile J, de la Iglesia HO, Scheuer T, Catterall WA (2015). Sleep impairment and reduced interneuron excitability in a mouse model of Dravet syndrome. Neurobiol. Dis..

[30] Han S, Yu FH, Schwartz MD, Linton JD, Bosma MM, Hurley JB, Catterall WA, de la Iglesia HO (2012). Na(V)11 channels are critical for intercellular communication in the suprachiasmatic nucleus and for normal circadian rhythms. Proc. Natl. Acad. Sci. U.S.A..

[31] Han S, Tai C, Westenbroek RE, Yu FH, Cheah CS, Potter GB, Rubenstein JL, Scheuer T, de la Iglesia HO, Catterall WA (2012). Autistic-like behaviour in Scn1a^+/^^−^ mice and rescue by enhanced GABA-mediated neurotransmission. Nature.

[32] Rubinstein M, Han S, Tai C, Westenbroek RE, Hunker A, Scheuer T, Catterall WA (2015). Dissecting the phenotypes of Dravet syndrome by gene deletion. Brain.

[33] Ito S, Ogiwara I, Yamada K, Miyamoto H, Hensch TK, Osawa M, Yamakawa K (2013). Mouse with Nav1.1 haploinsufficiency, a model for Dravet syndrome, exhibits lowered sociability and learning impairment. Neurobiol. Dis..

[34] Kalume F, Westenbroek RE, Cheah CS, Yu FH, Oakley JC, Scheuer T, Catterall WA (2013). Sudden unexpected death in a mouse model of Dravet syndrome. J. Clin. Investig..

[35] Auerbach DS, Jones J, Clawson BC, Offord J, Lenk GM, Ogiwara I, Yamakawa K, Meisler MH, Parent JM, Isom LL (2013). Altered cardiac electrophysiology and SUDEP in a model of Dravet syndrome. PLoS One.

[36] Tatsukawa T, Ogiwara I, Mazaki E, Shimohata A, Yamakawa K (2018). Impairments in social novelty recognition and spatial memory in mice with conditional deletion of Scn1a in parvalbumin-expressing cells. Neurobiol. Dis..

[37] Campbell JD, Whittington MD, Kim CH, VanderVeen GR, Knupp KG, Gammaitoni A (2018). Assessing the impact of caring for a child with Dravet syndrome: Results of a caregiver survey. Epilepsy Behav..

[38] Jensen MP, Liljenquist KS, Bocell F, Gammaitoni AR, Aron CR, Galer BS, Amtmann D (2017). Life impact of caregiving for severe childhood epilepsy: Results of expert panels and caregiver focus groups. Epilepsy Behav..

[39] Connolly MB (2016). Dravet syndrome: Diagnosis and long-term course. Can. J. Neurol. Sci..

