# Supplementary material for: Clinical spectrum and the comorbidities of Dravet syndrome in Taiwan and the possible molecular mechanisms
Source: Sci Rep. 2021 Oct 12;11:20242. doi: 10.1038/s41598-021-98517-4 (PMC8511137; doi:10.1038/s41598-021-98517-4)
Supplement: Supplementary file 1 — Supplementary Tables. [file 41598_2021_98517_MOESM1_ESM.docx]

**Clinical spectrum and the comorbidities of Dravet Syndrome in Taiwan and the possible molecular mechanisms**

Chia-Hsuan Huang^1^, Pi-Lien Hung^2^, Pi-Chuan Fan^3^, Kuang-Lin Lin^4^, Ting-Rong Hsu^5^, I-Jun Chou^4^, Che-Sheng Ho^6^, I-Ching Chou^7^, Wei-Sheng Lin^5^, Inn-Chi Lee^8^, Hueng-Chuen Fan^9^, Shyi-Jou Chen^10^, Jao-Shwann Liang^11^, Yi-Fang Tu^12^, Tung-Ming Chang^13^, Su-Ching Hu^14^, Lee-Chin Wong^3,14^, Kun-Long Hung^15^, Wang-Tso Lee^3,*^

^1^Division of Pediatric Neurology, Department of Pediatrics, National Taiwan University Hospital Yunlin Branch, Yunlin County, Taiwan

^2^Department of Pediatric Neurology, Chang Gung Memorial Hospital-Kaohsiung, Kaohsiung, Taiwan

^3^Department of Pediatrics, National Taiwan University Hospital and National Taiwan University College of Medicine, Taipei, Taiwan

^4^Division of Pediatric Neurology, Chang Gung Children's Hospital and Chang Gung Memorial Hospital, Chang Gung University College of Medicine, Taoyuan, Taiwan

^5^Department of Pediatrics, Taipei Veterans General Hospital, Taipei, Taiwan

^6^Department of Pediatrics, Mackay Memorial Hospital, Taipei, Taiwan

^7^Division of Pediatrics Neurology, China Medical University, Children's Hospital, Taichung, Taiwan

^8^Institute of Medicine, School of Medicine, Chung-Shan Medical University, Taichung, Taiwan

^9^Department of Pediatrics, Tungs' Taichung Metroharbor Hospital, Taichung, Taiwan

^10^Department of Pediatrics, Tri-Service General Hospital, National Defense Medical Center, Taipei, Taiwan

^11^Department of Pediatrics, Far Eastern Memorial Hospital, New Taipei City, Taiwan

^12^Department of Pediatrics, National Cheng Kung University Hospital, College of Medicine, National Cheng Kung University, Tainan, Taiwan

^13^Department of Pediatric Neurology, Changhua Christian Children's Hospital Changhua, Taiwan

^14^Department of Pediatrics, Cathay General Hospital, Taipei, Taiwan

^15^ Department of Pediatrics, Fu-Jen Catholic University Hospital, Fu-Jen Catholic University, New Taipei City, Taiwan

Supp Table 1. Influence of DS caregiving on Oberst Caregiving Burden Scale domain

| Distribution of responses | Response (n=38) | | | | | | |  |
| --- | --- | --- | --- | --- | --- | --- | --- | --- |
|  | No problem | | Some problems | | Extreme problem | | |  |
| Additional household tasks | 8 | 21% | 25 | 66% | 5 | 13% | |  |
| Symptom observation | 9 | 24% | 28 | 74% | 1 | 3% | |  |
| Further medical plan | 9 | 24% | 24 | 63% | 5 | 13% | |  |
| Financial problem | 13 | 34% | 18 | 47% | 7 | 18% | |  |
| Medical or nursing treatments | 13 | 34% | 20 | 53% | 5 | 13% | |  |
| Medication use | 14 | 37% | 21 | 55% | 3 | 8% | |  |
| Patient care | 16 | 42% | 19 | 50% | 3 | 8% | |  |
| Mobility | 19 | 50% | 18 | 47% | 1 | 3% | |  |
| Supp Table 2. Most common caregiver concerns | | | | | | |  | |
| **Concern** | | | | | | | **n=38** | |
| Lack of independence/constant care | | | | | | | 23(60.5%) | |
| Seizure control | | | | | | | 22 (57.9%) | |
| Speech/communication | | | | | | | 19 (50%) | |
| Sibling impacts/Long-term care when parents are gone | | | | | | | 19 (50%) | |
| Cognitive/developmental delay/regression | | | | | | | 11(28.9%) | |
| Behavioral issues including violence and autistic traits | | | | | | | 3(7.9%) | |
| SUDEP or death | | | | | | | 4(10.5%) | |
| Anxiety/depression/isolation | | | | | | | 2(5.2%) | |
| Finances | | | | | | | 7(18.4%) | |
| Medication side effects | | | | | | | 5(13.2%) | |
| Caregivers were asked to “List the top 3 concerns for the patient or family” after seizure control in open response. | | | | | | | | |
